# Ether vs. Chloroform

**Published:** 1874-01

**Authors:** 


Not long since, upon the occasion of the meeting, in Great Britain, of an International Ophthalmological Congress, Dr. B. Joy Jeffries, of Boston, called attention anew to the efficacy and superior safety of ether as an anæsthetic in surgery. Subsequently the subject has given rise to much experiment, and no little discussion in professional circles across the waters. It has, likewise, drawn attention again to the comparative safety of the two principal anæsthetics—ether and chloroform—in use in this country. It is well that it is so; for if the claim made, and so often and persistently reiterated by our brethren in Boston be, indeed, founded in truth and sober fact, then is the subject worthy of all the attention which it is now receiving.

We re-publish, elsewhere, from the pen of Dr. Henry J.

Bigelow, Surgeon to the Massachusetts General Hospital, and a very competent authority upon the subject, a series of practical suggestions upon the anæsthetic use of ether, drawn from the very large experience of that surgeon in the hospital under his care, where ether is the exclusive anæsthetic in use.

It was formerly claimed for ether, by its Boston champions, that it had never been known to produce death. Whilst, of late, this very high ground of advocacy has been partially yielded, still quite enough is yet claimed for its safety, if adequately sustained, to entitle it to a decided preference over chloroform for anæsthetic uses.

If we are correctly informed, ether has taken the place of chloroform in all the great hospitals and college clinics of Philadelphia and New York, and we think we do not misstate the facts, when we say that no physician or surgeon can, at this day, administer chloroform to anæsthesia in the city of Boston, except at his own peril, and under the frown of his professional brethren. Under this state of facts, it is but an act of common prudence for practitioners, everywhere, who are in the habit still of using chloroform, and this alone, for anæsthetic purposes to review carefully and well the ground upon which they stand.

In this connection, it may not be amiss to call attention to two or three points connected with the exhibition of anæsthetics. It has been often observed that deaths consequent upon anæsthesia are found, out of all due proportion, among those who are undergoing comparatively trivial operations, e.g., extraction of teeth, etc. It has been asserted that the pain consequent upon slight and transitory operations, fails to counteract the depressing influences of the anæsthetic, as the more severe and protracted ordeal of the graver operations is believed to do. But it has likewise been suggested—and we believe with important and cogent reasoning—that the special mortality in these cases is due, at least in greater part, to the erect or semi-erect position of the body of the patient, whereby fatal syncope is induced, in consequence of weakened action of the heart and the gravity of the blood, causing failure of adequate cerebral supply.

Besides, in the sitting posture, as usually practiced, an obstacle is offered, by the pressure of the abdominal organs upwards in the flexed state of the spinal column, to the free descent of the diaphragm and perfect and full respiration. There can be no real and insurmountable necessity for the exhibition of anæsthetics in the erect or sitting position of the patient; and the sooner the increased danger thereby incurred is duly recognized by the profession, and acted upon, the better for the cause of humanity. We can assert, with the confidence given by no little experience, that the extraction of teeth, under ether or chloroform, can be effected with as much, and sometimes even more, facility in the reclining position; whilst the nervous shock to a delicate lady not anæsthetized, is much better borne.

We would call attention, too, to the very great differences in specific gravity and volatility between chloroform and ether; in consequence of which a mixture of the two liquids does not yield a vapor of uniform density and composition; being at first supercharged with ether, and afterwards with chloroform.

When it is desired to combine the anæsthetic effects of the two, it is probable that more uniform results would follow the alternate administration of each, as might be desired. In the use of ether, we have occasionally found it desirable to deepen a little the effect of the ether by a few drops of chloroform upon the sponge. When alarming symptoms appear, in the use of chloroform, it should be recollected that the density of its vapor is very great, and suitable efforts should be made to displace it as far as possible from the air-tubes, by lowering the head and compressing the chest, and by promptly diluting it with air by artificial respiration.

